# The impact of common variants on gene expression in the human brain: from RNA to protein to schizophrenia risk

**DOI:** 10.1101/2023.06.04.543603

**Published:** 2023-10-10

**Authors:** Qiuman Liang, Yi Jiang, Annie W. Shieh, Dan Zhou, Rui Chen, Feiran Wang, Meng Xu, Mingming Niu, Xusheng Wang, Dalila Pinto, Yue Wang, Lijun Cheng, Ramu Vadukapuram, Chunling Zhang, Kay Grennan, Gina Giase, The PsychENCODE Consortium, Kevin P White, Junmin Peng, Bingshan Li, Chunyu Liu, Chao Chen, Sidney H. Wang

**Affiliations:** 1 MOE Key Laboratory of Rare Pediatric Diseases & Hunan Key Laboratory of Medical Genetics, School of Life Sciences, and Department of Psychiatry, The Second Xiangya Hospital, Central South University, Changsha, Hunan 410000, China.; 2 Department of Epidemiology and Biostatistics, Ministry of Education Key Laboratory of Environment and Health, School of Public Health, Tongji Medical College, Huazhong University of Science and Technology, Wuhan, Hubei 430000, China.; 3 Center for Human Genetics, The Brown foundation Institute of Molecular Medicine, The University of Texas Health Science Center at Houston, Houston, TX 77030, USA.; 4 School of Public Health and the Second Affiliated Hospital, Zhejiang University School of Medicine, Hangzhou, Zhejiang 310058, China.; 5 Department of Molecular Physiology and Biophysics, Vanderbilt Genetics Institute, Vanderbilt University, Nashville, TN 37232, USA.; 6 Department of Structural Biology, Department of Developmental Neurobiology, Center for Proteomics and Metabolomics, St. Jude Children’s Research Hospital, Memphis, TN 38105, USA.; 7 Department of Genetics, Genomics, and Informatics, University of Tennessee Health Science Center, Memphis, TN 38163, USA.; 8 Department of Psychiatry, and Seaver Autism Center for Research and Treatment, Icahn School of Medicine at Mount Sinai, New York, NY 10029, USA.; 9 Department of Genetics and Genomic Sciences, and Icahn Institute for Data Science and Genomic Technology, Icahn School of Medicine at Mount Sinai, New York, NY 10029, USA.; 10 The Mindich Child Health and Development Institute, Icahn School of Medicine at Mount Sinai, New York, NY 10029, USA.; 11 Friedman Brain Institute, Icahn School of Medicine at Mount Sinai, New York, NY 10029, USA.; 12 Department of Electrical and Computer Engineering, Virginia Polytechnic Institute and State University, Arlington, VA 22203, USA.; 13 Institute for Genomics and Systems Biology, University of Chicago, Chicago, IL 60637, USA.; 14 Department of Psychiatry, The University of Texas Rio Grande Valley, Harlingen, TX 78550, USA.; 15 Department of Neuroscience and Physiology, SUNY Upstate Medical University, Syracuse, NY 13210, USA.; 16 Department of Psychiatry, SUNY Upstate Medical University, Syracuse, NY 13210, USA.; 17 The Feinberg School of Medicine, Northwestern University, Chicago, IL 60611, USA.; 18 Department of Biochemistry, Yong Loo Lin School of Medicine, National University of Singapore, Singapore 117596, Singapore.; 19 School of Psychology, Shaanxi Normal University, Xi’an, Shaanxi 710062, China.; 20 Furong Laboratory, Changsha, Hunan 410000, China.; 21 National Clinical Research Center for Mental Disorders, The Second Xiangya Hospital, Central South University, Changsha, Hunan 410000, China.; 22 National Engineering Research Center of Personalized Diagnostic and Therapeutic Technology, Central South University, Changsha, Hunan 410000, China.

## Abstract

**Background:**

The impact of genetic variants on gene expression has been intensely studied at the transcription level, yielding in valuable insights into the association between genes and the risk of complex disorders, such as schizophrenia (SCZ). However, the downstream impact of these variants and the molecular mechanisms connecting transcription variation to disease risk are not well understood.

**Results:**

We quantitated ribosome occupancy in prefrontal cortex samples of the BrainGVEX cohort. Together with transcriptomics and proteomics data from the same cohort, we performed cis-Quantitative Trait Locus (QTL) mapping and identified 3,253 expression QTLs (eQTLs), 1,344 ribosome occupancy QTLs (rQTLs), and 657 protein QTLs (pQTLs) out of 7,458 genes quantitated in all three omics types from 185 samples. Of the eQTLs identified, only 34% have their effects propagated to the protein level. Further analysis on the effect size of eQTLs showed clear post-transcriptional attenuation of eQTL effects.

To investigate the biological relevance of the attenuated eQTLs, we identified 70 expression-specific QTLs (esQTLs), 51 ribosome-occupancy-specific QTLs (rsQTLs), and 107 protein-specific QTLs (psQTLs). Five of these omics-specific QTLs showed strong colocalization with SCZ GWAS signals, three of them are esQTLs. Using S-PrediXcan we identified 74 SCZ risk genes, 34% of which were novel, and 67% of these risk genes were replicated in a MR-Egger test. Notably, the majority (52) of these risk genes were identified using eQTL data and 70% of these SCZ-risk-gene-driving eQTLs show little to no evidence of driving corresponding variations at the protein level.

**Conclusion:**

The effect of eQTLs on gene expression in the prefrontal cortex is commonly attenuated post-transcriptionally. Many of the attenuated eQTLs still correlate with SCZ GWAS signal. Further investigation is needed to elucidate a mechanistic link between attenuated eQTLs and SCZ disease risk.

## Introduction

Complex diseases such as neuropsychiatric disorders are multi-factorial with genetic components (1, 2). Large scale Genome-Wide Association Studies (GWAS) have uncovered thousands of disease associated loci, signaling a promising era ahead for causal variant identification (3). However, efforts in fine mapping these disease risk loci often narrowed down the underlying causal signals to non-coding regions of the genome (4–6). Regulatory variants in such non-coding regions are therefore the prime candidates for driving the genetic risk of disease etiology. Consequently, integrating gene expression information to pinpoint causal variants or to identify risk genes has become a staple of genetic studies of complex diseases (7), with multiple consortia efforts facilitating large scale gene expression profiling and regulatory element mapping (8–10). Many powerful methods, such as coloc, PrediXcan, SMR/HEIDI, to name a few, have also been developed to leverage gene expression information for fine mapping GWAS signals or for identifying underlying risk genes (11–13).

Schizophrenia (SCZ) is a psychiatric disorder affecting ~1% of the world-wide population (14). The heritability of SCZ has been estimated at between 60% to 80% indicating a strong genetic component (15, 16). Accordingly, recent SCZ GWAS study reported by Trubetskoy et al. identified 287 significant risk loci and prioritized 120 risk genes using functional genomic data (17). The use of RNA-Seq data from the brain was instrumental for risk gene prioritization by Trubetskoy et al.; however, information from downstream gene regulation processes, such as translation rate and protein abundance, was either not utilized or unavailable.

Measuring transcriptional changes as a proxy for gene activity has a long history in molecular biology (18). In the context of human genetics, buffering of downstream effects of genetic variants impacting gene expression (i.e. an eQTL) has been shown to be prevalent (19). In addition, QTLs specific to protein level have also been reported (19–21). Together, these observations indicated the importance and potential benefits of including downstream omics types, such as proteomics data, as information sources for fine mapping disease regulatory variants. Indeed, recent studies using multi-omics approaches have demonstrated increased power for risk gene identification among other benefits (22, 23). Of note, our recent work on genetic variants associated with protein level in prefrontal cortex of the human brain indicated the extent of contribution from non-synonymous coding variants to changes at the protein level, and the utility of these protein QTL variants in prioritizing GWAS risk genes for psychiatric disorders (21).

Another potential benefit of taking a multi-omics approach for identifying disease risk genes rests in the potential to dissect the fine details of regulatory mechanisms driving the disease-genotype association. Having relevant datasets to illuminate the origin and propagation of genetic impact could potentially arrive at a conclusion of the driver regulatory process for a risk gene. Ribo-seq is a technology that can be used to collect relevant data to fill in the gap between transcript and protein expression. By adapting RNA-Seq to a ribosome footprinting method, ribo-seq provides transcriptome-wide quantification of ribosome occupancy (24, 25), which can serve as a proxy for the amount of active translation synthesizing proteins from each mRNA transcript. When analyzed in conjunction with RNA-Seq and quantitative proteomics data, ribo-seq enables identification of translational and post-translational regulatory events (19, 26), both major steps defining the human genetics aspect of the Central Dogma of molecular biology.

As a part of the consortium efforts to improve our understanding of the genetic basis of neuropsychiatric disorders (27), we generated multiple data modalities that included SNP genotyping, RNA-Seq, ribo-seq, and proteomics of postmortem cortical tissue samples of the BrainGVEX cohort, which altogether covered multiple omics levels from DNA, transcript to protein. In conjunction with the quantitative proteomics and transcriptome profiling results that we previously published (21, 28), here we integrated ribo-seq data as our operational definition for protein translation based on the transcriptome to make it a true multi-omics investigation. Our results reveal regulatory properties of common variants in the human brain and their utility in identifying the regulatory processes driving disease risk for schizophrenia. It offers an opportunity from a population angle to dissect and appreciate the regulatory information flow in the biological processes associated with the Central Dogma. Additional “rules” including information attenuation and modification regulation in the process are recognized.

## Results

### Measuring transcriptome-wide ribosome occupancy level in prefrontal cortex of adult human brain to quantify the level of protein translation

To investigate regulatory impact of genetic variants on protein translation in the prefrontal cortex of the human brain, we performed ribosome profiling on 211 prefrontal cortex samples from the BrainGVEX collection. In total we collected ~62 billion ribosome profiling reads. Consistent with the expected ribosome footprint size, we found the average insert size of our ribo-seq libraries to range between 27.4 and 29.5 nucleotides. Similar to prior published studies (29), we found on average 74 % of unwanted reads from ribosomal RNA, tRNA, and snoRNA, which contributed no information to the translation of protein coding genes. After removing these unwanted reads, we obtained an average of 30.3 million uniquely mapped informative reads per sample (inter-quartile range: 20.5 ~ 37.6 million reads). When focusing our analysis on the informative reads, we found our ribo-seq data to have ~3 times higher proportion of exon reads than that of the total RNA-Seq data collected from the same individuals using an rRNA-depletion method (28). When visualized in aggregate across annotated coding genes, we found our ribo-seq data to show strong sub-codon periodicity at the expected positions (Fig. S1). High proportion of exon reads and strong sub-codon periodicity reflect the enrichment of footprints from ribosomes actively engaged in translating mature mRNA and indicates the quality of the dataset.

### Multi-omics cis-QTL mapping identified candidate regulatory variants and revealed translational and post-translational attenuation of eQTL effects

**Fig. 1.**
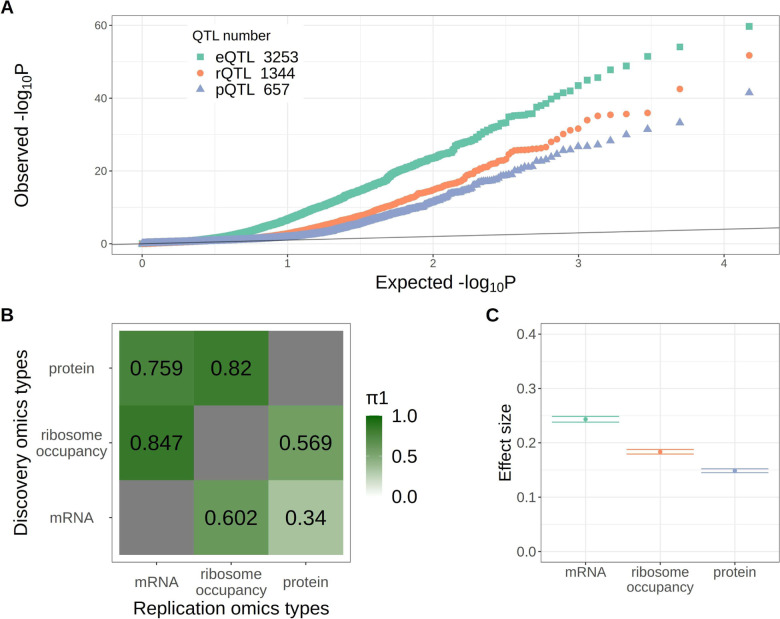
Genetic regulation of gene expression in the human brain. **(A)** P-value quantile-quantile plot between the observed (Y-axis) and the expected based on null distribution (X-axis). The black line indicates the expected distribution of p values when there are no real QTL signals. The number of cis-QTLs (i.e. the most significantly associated SNP for each gene) identified at 10% FDR is labeled in the top left inset. **(B)** Replication rate between QTL types. Proportions of QTLs replicated in the other two omics types are listed in the 3X3 matrix. Each row is a discovery omics type and each element of the row correspond to the proportion QTL signals replicated in the omics type specified by the column label. For example, only 34% of the eQTL signals were replicated in the protein data. **(C)** Effect size of CMC eQTL SNPs in BrainGVEX data. Mean and 95% confidence interval of absolute per allele effect across all CMC eQTL SNPs that were also analyzed in the BrainGVEX union set is shown.

To identify variants associated with inter-individual expression differences, we perform cis-QTL mapping for each omics type independently. Using the full dataset (i.e. 416 RNA-Seq samples, 195 ribo-seq samples, and 268 proteomics samples, which are coupled with the corresponding genotype data), we identified 12,411 eQTLs (out of 16,540 genes we deem sufficiently quantitated), 2,849 rQTLs (out of 14,572 genes), and 1,036 pQTLs (out of 8,013 genes) at FDR < 0.1. The majority of the eQTLs identified here were replicated in the prefrontal cortex RNA-Seq data from the GTEx consortium (Table S1). Intriguingly, we found drastic differences between omics types in the numbers of QTLs mapped, suggesting that some of the eQTL effects do not propagate all the way to the protein level. However, the differences in the number of genes tested between omics types and the differences in sample size make the comparison challenging to interpret. To better compare the effects of genetic regulation between multiple data modalities, we identified 185 samples with 7,458 genes that were sufficiently quantitated across all three omics types. Using this unified dataset, we found 3,253 eQTLs, 1,344 rQTLs, and 657 pQTLs at FDR <0.1 (Fig. 1A, Fig. S2, Table S2, S3, S4). Similar to the results derived from the full dataset, using the unified dataset we found fewer significant QTLs as we moved downstream the Central Dogma of molecular biology.

A challenge in comparing between the numbers of QTLs identified from each omics type rests in the fact that not all true effects were identified. Tests replicating QTLs identified from one omics type in the other omics types can better capture the proportion of genetic effects shared between QTL types. We performed replication tests using π1 estimates from the qvalue method (30). Overall, we found high proportion of QTLs replicated in other omics types (Fig. 1B). However, when considering the replication rates with the direction of genetic information flow, we found asymmetric replication rates, with the downstream omics types to replicate less than the upstream omics types. More specifically, we found 84.7% of the rQTLs were replicated at the transcript level, but only 60.2% of the eQTLs were replicated at the ribosome occupancy level. Moreover, while 75.9% of the pQTLs were replicated at the transcript level, only 34.0% of the eQTLs were replicated at the protein level (Fig. 1B). The lower percentages of eQTLs and rQTLs replicated at the protein level indicate potential effect attenuation (i.e. either the inter-individual variation in gene expression becoming smaller and therefore harder to detect or a lack of such effect in the downstream omics types). Interestingly, in addition to the effect attenuation at the protein level, which was previously reported for lymphoblastoid cell lines (LCLs) (19), here, using brain samples, we found further asymmetry between eQTL and rQTL replication, indicating effect attenuation at the level of translation. A similar asymmetry in proportion replicated between upstream and downstream omics types was observed when using a direction-aware cutoff-based approach across a wide range of significance cutoffs used to define replication rates (Fig. S3).

While our replication tests revealed a trend of effect attenuation for eQTL variants in the downstream phenotypes (Fig. 1B), these same observations could alternatively be explained by differences in statistical power between technologies. An independent piece of evidence that is not sensitive to measurement precision is needed to reach a solid conclusion. Using eQTLs independently identified from prefrontal cortex samples by the CommonMind Consortium (CMC) (31), we avoid the ascertainment bias for large effect size eQTLs identified from our dataset and can therefore directly compare the effects size of eQTL variants between the three omics types in our dataset. A similar approach was successfully implemented to address this power issue in previous work in LCLs (19). Using 5,915 CMC eQTLs that were also quantitated in our dataset, we found the eQTL effects on the transcript expression level to be significantly larger than their effects on ribosome occupancy level (per allele log2 fold differences: mRNA 0.2433 [95% CI= 0.2381~0.2486] vs. ribosome occupancy 0.1836 [95% CI= 0.1794~0.1878]), which were in turn significantly larger than their effects on protein level (per allele log2 fold differences: 0.1486 [95% CI= 0.1451~0.1522]) (Fig. 1C, t-test P < 2.2e^−16^ for all pairwise comparisons). By focusing on the effect sizes of independently identified eQTLs, our results strongly support the presence of downstream mechanisms attenuating eQTL effects both at the ribosome occupancy level (translationally) and at the protein level (post-translationally). Moreover, for the CMC eQTLs, we found translational regulation to account for more effect size reduction than post-translational regulation (Fig. 1C).

### Identifying omics specific QTLs and their signal colocalization with schizophrenia GWAS

The prevalent effect size reduction of eQTLs raised the question of the relevance of these genetic regulation at the organismal level. Because most cellular tasks are executed by proteins, the genetic regulatory effects not reaching the protein level are less likely to have an impact on organismal traits. To answer this question, we set out to investigate the relevance of different QTL types in SCZ. More specifically, we aim to identify expression specific QTLs (i.e. genetic variants that impact transcript level of the linked genes but not the downstream ribosome occupancy level nor protein level) that colocalize with SCZ GWAS.

Consistent with prior reports (32), using our full dataset we found significant proportion of SCZ heritability to be mediated by gene expression. By performing mediated expression score regression (MESC) (32) on summary statistics from the Trubetskoy et al. SCZ GWAS (17), we found our eQTLs to mediate 7.09%, rQTLs to mediate 4.06%, and pQTLs to mediate 2.17% of SCZ heritability (Table S5). After establishing the relevance for each of the three QTL types in SCZ, we next sought to identify omics-specific QTLs, in order to further evaluate their relevance in driving SCZ risk. Because regulation of a gene is often modulated by multiple genetic variants, to evaluate the consequence of overall cis-QTL impact on gene expression, we use PrediXcan to estimate aggregate genetic regulatory effects for each gene. To distinguish between genes that have QTL effects shared across multiple omics types and genes that have omics-specific QTL effects, for each omics type we built PrediXcan models with or without regressing out the other omics types and computed the correlation between the imputed expression from the two models. We termed this correlation R_c_. To identify R_c_^2^ values that reflect significant sharing between omics types, we permuted sample labels to emulate conditions of no real correlation between the three omics types, in order to generate empirical null distributions. Using both a false discovery rate (FDR), which is calculated based on the empirical null, and an effect size cutoff based on R_c_^2^, we defined a set of shared QTL genes and three sets of omics-specific QTL genes. At 10% FDR, we defined shared QTL genes by further requiring the R_c_^2^ to be smaller than 0.5. Using these criteria, from the 1,354 genes that passed the minimum PrediXcan criteria for being included in this analysis (see details in methods), we identified 295 shared QTL genes that have QTL effects shared between at least two omics types. For genes that failed to reject null at the 10% FDR cutoff (i.e. potentially omics-specific) we further set a conservative R_c_^2^ > 0.9 cutoff to define omics-specific QTLs for those QTLs that did not change after regressing out effects from other omics types. Using these criteria, we found 70 esQTL (mRNA-specific QTL) genes, 51 rsQTL (ribosome occupancy-specific QTL) genes, and 107 psQTL (protein-specific QTL) genes (Fig. S4, Table S6).

**Fig. 2.**
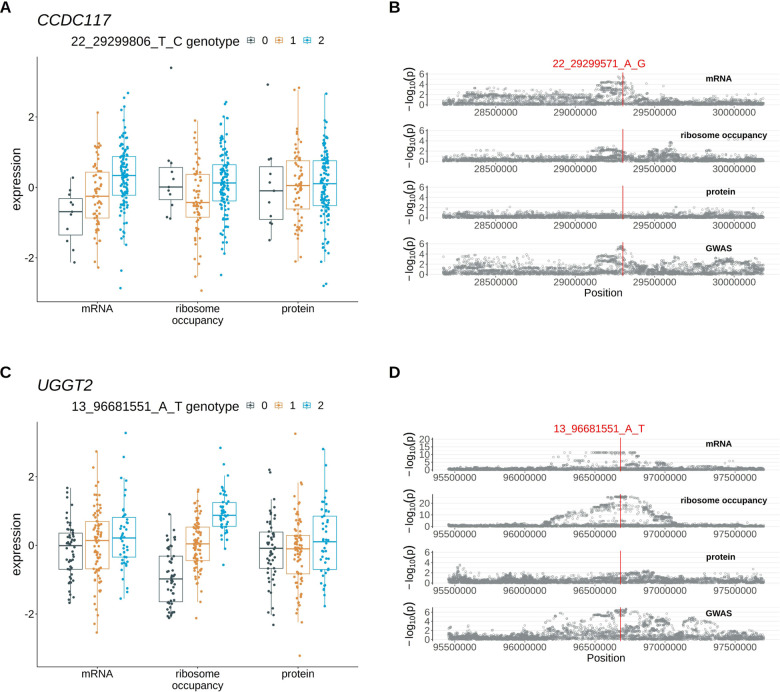
Signal colocalization between schizophrenia GWAS and omics-specific QTLs. **(A, C)** Boxplots summarizing normalized gene expression level stratified by QTL genotypes for *CCDC117* esQTLs (A) and *UGGT2* rsQTLs (C). **(B, D)** Manhattan plots showing p value distribution for each QTL type and schizophrenia GWAS for the 1Mb QTL mapping window flanking *CCDC117* (B) and *UGGT2* (D). The red line indicates the position of the lead colocalization SNP between omics-specific QTLs and schizophrenia GWAS.

To investigate the relevance of omics-specific QTL genes in SCZ, we performed summary statistics-based signal colocalization between QTL signals and SCZ GWAS signals. Using *coloc* with default prior (11), at a posterior probability cutoff of 70% for the signal colocalization hypothesis, we found esQTLs of 3 genes, *CCDC117, GATAD2A,* and *JAKMIP2* to colocalize with SCZ GWAS signals at loci *22q12.1*, *19p13.11,* and *5q32*, respectively (Fig. 2A, Fig. 2B). In addition, we found rsQTLs of *UGGT2* to colocalize with SCZ GWAS signals at locus *13q32.1* (Fig. 2C, Fig. 2D) and psQTLs of *P2RX7* to colocalize with SCZ GWAS signals at locus *12q24.31*. On the other hand, for shared QTL genes, we found the eQTLs of 6 genes and the rQTLs of 1 gene to colocalize with SCZ GWAS signals. In summary, we identified strong signal colocalization with SCZ GWAS from both shared QTLs and omics-specific QTLs, at comparable proportions (i.e. 7 from 295 of shared vs. 5 from 228 of omics-specific). This indicates that the omics-specific QTLs are equally important in explaining SCZ GWAS signals.

### Functional genomics identification of Schizophrenia risk genes

**Fig. 3.**
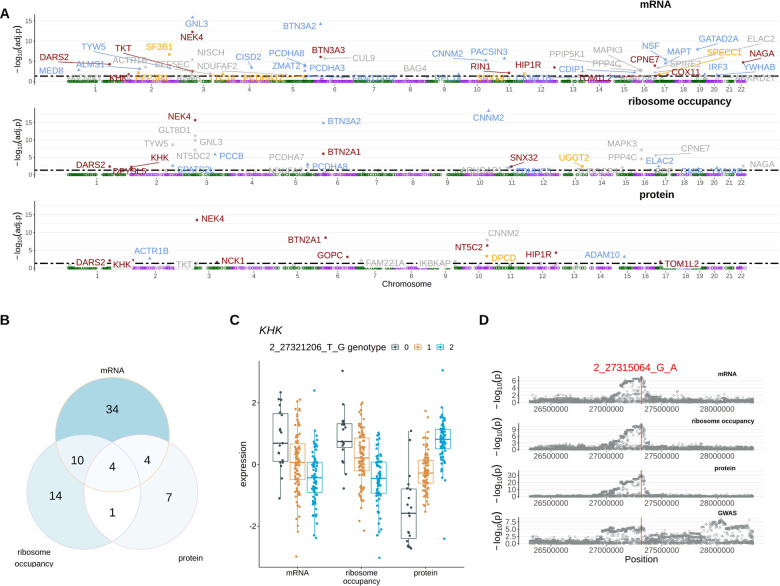
Schizophrenia risk genes identified from each of the three omics types RNA-Seq (mRNA), ribo-seq (ribosome occupancy), and proteomics (protein) using S-PrediXcan. **(A)** Manhattan plots showing significance level (i.e. −log10 FWER from S-PrediXcan) of gene-schizophrenia association across the genome for genes that pass the 5% FWER significance cutoff. The black horizontal dotted line indicated the significance cutoff. Risk genes are color-coded according to the MR test results. Grey asterisks mark the risk genes that failed the two-sample MR tests; dark red solid circle marks the risk genes that pass both one-sample MR tests (passing both one-sample MR tests suggest that transcriptionally regulated protein level differences between individuals drives the disease risk); blue triangle marks the risk genes that pass one of the two one-sample MR tests; orange rectangle marks the risk genes that failed both one-sample MR tests. **(B)** Venn diagram illustrating the number and corresponding percentage of overlapping risk genes between omics types. **(C)** Boxplots summarizing normalized gene expression level stratified by eQTL genotypes for *KHK*. **(D)** Manhattan plots showing p value distribution for each QTL type and schizophrenia GWAS for the 1Mb QTL mapping window flanking *KHK*. The red line indicates the position of the lead colocalization SNP between eQTLs and schizophrenia GWAS.

To further investigate the relevance of attenuated eQTLs in SCZ risk, we next took a complementary approach by first identifying risk genes from each omics type separately and then investigating the relevant regulatory processes driving SCZ risk. Following the observed percentages of SCZ heritability mediated by gene regulation found in our full dataset, here using the same GWAS we focused our effort to identify risk genes for SCZ based on our unified multi-omics dataset using S-PrediXcan (33). At 5% family-wise error rate, we found 52, 29, and 16 SCZ risk genes, respectively from RNA-Seq, ribo-seq, and proteomics data (Fig. 3A, Fig. S5; note that the color and shape code of this figure will become relevant in the next results section). Among them only four genes, *NEK4*, *KHK*, *CNNM2* and *DARS2* were consistently identified as SCZ risk genes from all three omics types (Fig. 3B). The majority (74.3%) of the risk genes were identified only from one of the three omics types. This limited sharing in risk gene identification between omics types is in clear contrast to the amount of signal sharing found between QTL types (Fig. 1B).

Among the 74 risk genes we identified using S-PrediXcan (i.e. the union between the risk genes identified from each of the three omics types), 44 have previously been reported in GWAS studies as either the mapped genes or as one of the nearby genes under the GWAS peak (Table S7) (17, 34–36). Of these previously reported genes, 27 matched the mapped genes while the remaining 17 nominated an alternative candidate gene for each GWAS locus (Note that for some of these 17 loci, the original GWAS signals were mapped to an intergenic region). Comparing our results to other published SCZ risk gene identification studies, we found 15 matched to the risk genes identified by Giambartolomei et al., which used RNA-Seq and DNA methylation data from prefrontal cortex of the human brain (22) and 12 matched to the 120 prioritized SCZ risk genes from Trubetskoy et al., which used colocalization with eQTL and Hi-C data (17). On the other hand, for 25 of our 74 risk genes (33.8%), we found no match to the known risk gene list (Table S8), which we compiled based on previous GWAS and functional genomics risk gene identification studies (17, 22, 34–36). These “no match” novel risk genes have relatively weak SCZ GWAS signals and are therefore challenging to identify without the additional information provided in our multi-omics QTL dataset. For example, we found strong colocalization between a modest SCZ GWAS signal at *2p23.3* and all three types of molecular QTLs of the gene *KHK* (Fig. 3D). Intriguingly, the *KHK* pQTL is in opposite direction of the *KHK* eQTL and rQTL (Fig. 3C), suggesting a linked post-translational process regulating the protein level in addition to the transcriptional regulation. *KHK*, known as Ketohexokinase, plays a pivotal role in fructose metabolism and has been hypothesized to contribute significantly to sustaining neuronal function (37). Another novel SCZ risk genes, *BTN3A2*, belongs to the Butyrophilin Subfamily 3. Overexpression of *BTN3A2* has been observed to inhibit excitatory synaptic activity onto CA1 pyramidal neurons (38). *NSF*, N-Ethylmaleimide Sensitive Factor, which encodes a vesicle fusing ATPase, has been identified as a causal factor in intelligence traits (39).

### Analyses of multi-omics dataset reveal regulatory mechanisms of schizophrenia risk genes

While PrediXcan is a powerful tool for GWAS risk gene identification, it does not control for potential horizontal pleiotropy (40). To this end, we performed two-sample Mendelian Randomization (MR) with Egger regression to replicate the risk genes we identified using S-PrediXcan. Egger regression includes an intercept term, which can be used to evaluate the level of horizontal pleiotropy (41). For each risk gene we first used LD-clumping (42) to identify top SNPs from independent signals as strong genetic instruments (43). We then tested for the causal relationship between gene regulation (i.e. QTL signal) and SCZ (i.e. GWAS). We used an operational definition of a causal effect based on the MR test results (see Methods). At 10% FDR, of the 97 gene-by-omics combinations (i.e. a total of 74 risk genes including some discovered from more than one omics type), 67.0% (65/97) passed the MR test. Of those 33.0% that failed the MR test, 93.8% (30 out of 32) failed because of horizontal pleiotropy identified by the Egger intercept test (Table S9). A total of 52 genes were replicated in at least one of the three omics types. Similar, but stronger, causal effects were observed when using SuSiE (the Sum of Single Effects) (44) as an alternative approach to define instrument SNPs (Fig. S6). However, when using SuSiE for selecting instrument variables, 41 genes were tested because some of the risk genes had no fine mapped QTL SNPs according to SuSiE.

**Fig. 4.**
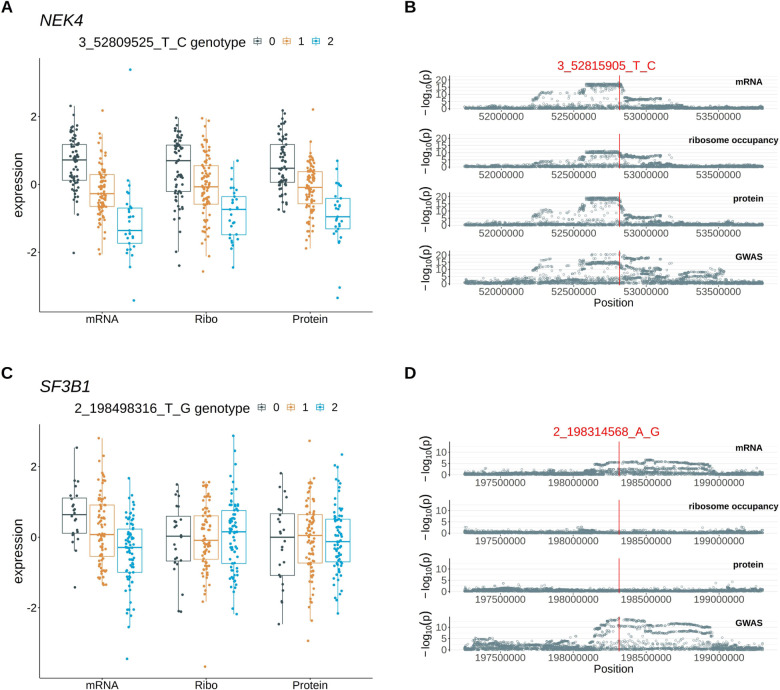
Signal colocalization between schizophrenia GWAS and eQTLs of example risk genes. **(A, C)** Boxplots summarizing normalized gene expression level stratified by QTL genotypes for *NEK4* eQTLs (A) and *SF3B1* eQTLs (C). **(B, D)** Manhattan plots showing p value distribution for each QTL type and schizophrenia GWAS for the 1Mb QTL mapping window flanking *NEK4* (B) and *SF3B1* (D). The red line indicates the position of the colocalization lead SNP between eQTLs and schizophrenia GWAS.

A key strength of using a multi-omics QTL approach to identify GWAS risk genes rests in the possibility of further narrowing down the potential regulatory mechanisms. To this end, we further examined the likely causal mechanisms for the 52 replicated SCZ risk genes using one-sample Mendelian Randomization to infer causality between QTL types. We focused our analysis on independently testing for causal effects between neighboring QTL types following the direction of information flow of the Central Dogma (i.e. mRNA -> ribosome occupancy, and ribosome occupancy -> protein). Here we used fine-mapped QTL SNPs identified from the exposure omics types (i.e. the upstream omics types) as instrument variables for one-sample MR analysis. Among the 52 two-sample MR replicated SCZ risk genes, we found 17 genes with significant causal effects both from eQTL to rQTL (i.e. the upstream pathway) and from rQTL to pQTL (i.e. the downstream pathway) (both-passed risk genes; Fig. 3A dark red solid circle datapoints). Significant causal effects detected from both pathways suggest transcriptionally regulated protein level differences as the potential mechanisms for these risk genes in SCZ etiology (see an example in Fig. 4A, Fig. 4B). On the other hand, 27 and 8 replicated risk genes have either significant causal effects only in one of the two pathways (single-passed risk genes; Fig. 3A blue triangle datapoints) or have no significant causal effects (none-passed risk genes; Fig. 3A orange rectangle datapoints), respectively.

Of note, for the 27 single-passed risk genes, we found significant causal effects only in the upstream pathway (mRNA -> ribosome occupancy). This asymmetry is reminiscent of the eQTL effect attenuation described in the prior sections. A failed test could indicate either a true lack of effect or a lack of statistical power. To take a closer look, we directly assessed the effect size, the noise level, and instrument strength of the one-sample MR test results. When comparing to the 17 both-passed risk genes, we found significantly smaller effect sizes both for failed tests of the 27 single-passed risk genes (only the downstream MR test results included, average 0.367 vs 0.055, t-test P < 4.9e^−5^, Fig. S7A) and the 8 none-passed risk genes (both upstream and downstream MR test results included, average 0.405 vs 0.033, t-test P < 4e^−12^, Fig. S8A). Note that in both cases the inter-quartile range of the estimated causal effects for the failed tests covered zero (Fig. S7A, Fig. S8A). On the other hand, for these same comparison groups, we found no significant difference in instrument strength (Fig. S7C, S8C, if anything slightly stronger instrument was observed in failed tests) and found only slightly higher noise level in the failed MR tests (Fig. S7B and S8B; t-test P = 0.026 and P= 0.013 respectively). The estimated small causal effects from the failed tests indicate that either we are observing weak effects that are obscured by the slightly elevated noise level (false negatives) or a lack of true causal effects, or a mixture of the two. In other words, some of these risk genes are likely to be driven by specific QTL types. Case in point, we found *SF3B1* to have similar patterns in p value distributions between eQTLs and SCZ GWAS but no clear QTL signals in either ribo-seq or proteomics data (Fig. 4C, Fig. 4D).

## Discussion

Using a panel of postmortem prefrontal cortex samples, we found clear evidence of post-transcriptional attenuation of eQTL effects in the human brain. Many of the differences found between individual brain transcriptomes were not present between individual brain proteomes. This observation echoes earlier work in HapMap lymphoblastoid cell lines (19) and extends the prior conclusion from *in vitro* cell lines to complex human tissues. Importantly, distinct from the earlier work in lymphoblastoid cell lines, which found translation to mostly track with transcription, we found clear attenuation of eQTL effect in ribo-seq data indicating that translational regulatory processes are involved in eQTL effect attenuation in the human brain. Prevalent translational attenuation of variant impact on transcriptional gene expression level has previously been reported in budding yeast (45, 46). However, results from follow up studies (47) appear to present an inconsistent picture. Here, using replication tests for individual eQTLs and testing for aggregate effect size of eQTLs independently identified from CMC, we provided strong evidences supporting prevalent translational attenuation of eQTL effects in the human brain. Although operating at the molecular level, our study remained observational. Omics-specific features could potentially confound the results. Future work replicating this observation and elucidating molecular mechanisms of translational attenuation of eQTL effects are needed to provide a clear understanding of the phenomenon.

Following this observation, our current work focuses on the important question of whether the attenuated eQTLs are functionally (biologically) relevant. We attempted to address this question by exploring the relevance of attenuated eQTLs in SCZ, a neuropsychiatric disorder that is highly heritable. We took two approaches to identify risk genes that have either omics-specific QTL signals or attenuated eQTLs. In the first approach, we used PrediXcan to aggregate variant impact at the gene level, in order to identify omics-specific risk genes. Our method is distinct from published work on omics-specific QTLs that took a SNP-based approach (19). Using a gene-based approach, we aimed to increase the interpretability of the results and decrease the challenge of the needle in the haystack problem. Indeed, our approach reduced our search space to 1,354 genes, and among these genes we identified 228 omics-specific QTL genes. By limiting our search to the most confident set of omics-specific QTL genes, we identified three clear examples of esQTLs that show strong colocalization with SCZ GWAS signals. At the same time, the limited number of discoveries put certain constraints on our ability to investigate the properties of esQTLs in the context of SCZ disease risk. It is expected that larger samples would reveal more esQTLs and enable deep mechanistic investigation.

In the second approach, we expanded our search by first identified risk genes from all quantitated genes using a TWAS approach, S-PrediXcan method. We then replicated the TWAS risk genes using two-sample Mendelian Randomization with Egger intercept tests. While the majority of the TWAS risk genes were replicated in two-sample Mendelian Randomization tests, of the minority that failed the MR tests most failed because of the Egger intercept test. Our results, therefore, clearly confirmed the need of controlling for potential horizontal pleiotropy in TWAS studies (41). About 34% of the risk genes that we identified here have not been previously reported as SCZ risk genes. These novel SCZ risk genes tend to have weak GWAS signals and are therefore challenging to identify without the help of functional genomics data (see Fig. 3D for an example). To identify causal regulatory mechanisms for each replicated risk gene, we further tested causality between QTL types using one-sample MR. We found 17 risk genes that are likely contributing to SCZ risk through transcriptionally regulated protein level. On the other hand, we also identify 8 genes that show no significant MR tests (candidate omics-specific risk genes) and 27 genes that have significant MR test results only from transcription to translation (candidate post-translationally-attenuated risk genes).

In essence, attempts to identify esQTLs (or any omics-specific QTLs) are dealing with the challenge of separating true negatives from false negatives. While we applied very conservative criteria to identify esQTLs and performed subsequent evaluations, such caveat is important to keep in mind when interpreting our results. Similarly, the interpretation of the failed MR tests is challenging. Our subsequent analyses looking at comparing instrument strength, noise level, and effect size between passed and failed MR tests, indicated comparable instrument strength, slightly elevated noise level in the failed tests, and clearly smaller effect size in the failed tests. In other words, the failed tests are reflecting either a smaller effect size obscured by noise in the data, or a true lack of causal effect, or possibly a mixture of both. In addition to the issues with false negative results in replication omics types, some of the omics-specific QTL discoveries could be false positives. Although we have good confidence with our FDR and FWER estimates, given that our test statistics for QTL mapping and risk gene identification are well calibrated (Fig. S2, S5), pleiotropy could introduce positive results from a different underlying cause (i.e. true effects on SCZ risk but false positive risk genes).

Power issues, however, do not explain the whole story. As was consistently observed throughout our study, when viewed in aggregate, we see clear effect size differences between omics types, both for QTLs and for causal effects from MR tests. These effect size estimates are not influenced by significance cutoffs and are not biased by power differences. Such general trends are also unlikely to simply be a result of spurious associations or made up entirely of false positives. Our results therefore bring forth an interesting mechanistic question: how do attenuated eQTL variants impact SCZ without changing protein levels? One interesting possibility is that the biologically relevant traits here are translation efficiency (i.e. protein synthesis rates) and protein turnover rates. For example, an association between genotype and translation efficiency could manifest in the form of an attenuated eQTL, where the differences in translation rate appears to offset the differences introduced at the transcript level, which in turn resulted in a lack of association between eQTL SNP genotype and ribosome occupancy level. In other words, the colocalization between an attenuated eQTL and GWAS signal could be reflecting a colocalization of GWAS signal with a translation efficiency QTL. Similarly a protein turnover QTL could also manifest in the form of a rQTL attenuated at the protein level. This hypothesis would predict additional linked regulatory variants (i.e. linked to the attenuated eQTLs) that impact translation efficiency or protein turnover rates. Moreover, the effect at the protein level may be missed at the pQTL due to technical issues specific to proteomics. We hope by presenting the current results, our findings can inspire future studies on this topic to understand the detailed regulation processes from DNA to RNA, protein and diseases.

## Materials and Methods

### Data sources

The SNP genotypes (21), RNA-Seq (28), and quantitative mass spectrometry (21) data generated for prefrontal cortex tissue samples of the BrainGVEX cohort were downloaded from the PsychENCODE Synapse portal (https://www.synapse.org/#!Synapse:syn5613798) (See Table S10 for a summary on the number of samples in each dataset and their respective overlap with the samples in the genotype data). The BrainGVEX cohort includes 420 Caucasians, 2 Hispanics, 1 African American, 3 Asian Americans, and 14 unknown-origin individuals. We also used PGC3 SCZ GWAS summary statistics data obtained from the Psychiatric Genomics Consortium (17).

### Ribosome profiling

Ribosome Profiling experiments were performed using Illumina’s TrueSeq Ribo Profile (Mammalian) Kit. TrueSeq Ribo Profile (Mammalian) Kit was developed for cell lines. We adapted the protocol to frozen tissue samples with MP Biomedicals^™^ FastPrep-24^™^ Classic Bead Beating Grinder and Lysis System. Specifically, 60–80 mg of frozen brain tissue was homogenized in Lysing Matrix D tubes containing 800 μl polysome lysis buffer. The Lysing tubes were placed on the FastPrep-24^™^ homogenizer set at 4.0 m/s with 20 s increments until no visible chunks of tissue remained. Tissue lysate was incubated on ice for 10 min followed by centrifugation at 20,000g at 4 °C for 10 minutes to pellet cell debris. Clarified lysate was subsequently used for ribo-seq library preparation following TrueSeq Ribo Profile (Mammalian) Kit instructions. Indexed libraries were pooled and then sequenced on an Illumina HiSeq 4000.

Note that the experimental protocol for TrueSeq Ribo Profile (Mammalian) Kit that we followed is a modified version of the previous ribo-seq protocol published by Ingolia and colleagues (25), and it has the following key modifications: Monosome isolation was performed using Sephacryl S400 spin columns (GE; 27–5140–01) on a tabletop centrifuge instead of ultra-high speed centrifugation in sucrose cushion. Ribosomal RNA depletion was carried out by using Ribo-Zero Magnetic Kits and this step is moved up to right after the monosomal RNA isolation step and before the Ribosome Protected Fragment gel isolation step.

### Data processing, gene expression quantification, and normalization

For RNA-Seq data, we obtained the FASTQ raw data from the PsychENCODE BrainGVEX project (https://www.synapse.org/#!Synapse:syn5613798). Then we used cutadapt (v1.12) to trim adapter for raw reads with code “cutadapt –minimum-length=5 –quality-base=33 -q 30 -a AGATCGGAAGAGCACACGTCTGAACTCCAGTCA -A AGATCGGAAGAGCGTCGTGTAGGGAAAGAGTGT”. Then we mapped trimmed reads onto GENCODE Release 19 (GRCh37.p13) genome with same version’s GTF by STAR (v2.4.2a). Then we used RSEM software (v1.2.29) to quantify the read counts for each gene (48). The cpm function in the R package “limma” was used to calculate the log-transformed counts per million (CPM). We filtered out the genes with CPM < 1 in more than 75% samples and the samples with network connectivity (49) z score < −5 (Fig. S9), which resulted in a total of 17,207 genes from 426 samples in the quantification table. We then used normalize.quantiles function in the R package “preprocessCore” (50) to normalize expression level for each sample. We used DRAMS software to detect and correct mixed-up samples (41), which resulted in final 423 samples.

For ribo-seq data, we used cutadapt (v1.12) to trim adapter for raw reads with code “cutadapt -a AGATCGGAAGAGCACACGTCT –quality-base=33 –minimum-length=25 –discard-untrimmed”. The trimmed reads were then mapped against a FASTA file of rRNA, tRNA, and snoRNA sequence downloaded from NCBI using bowtie2 (52) to filter out uninformative reads. The filtered reads were mapped to GENCODE Release 19 (GRCh37.p13) genome with corresponding transcript model GTF file using STAR (v2.4.2a). The uniquely mapped reads, as defined by the “NH:i:1” flag of the alignment files, were kept for subsequent analysis. We used the featureCounts function in the R package “subreads” (53) to calculate gene level read counts for ribosome occupancy. The cpm function in the R package “limma” was used to calculate log-transformed CPM value. We filtered out genes with CPM < 1 in more than 75% samples and the samples with network connectivity (49) z score < -3.5 (Fig. S10), which resulted in a total of 15,171 genes quantitated from 209 samples in the quantification table. We then used the normalize.quantiles function in the R package “preprocessCore” (50) to normalize ribosome occupancy level for each sample. We used DRAMS software to detect and correct mixed-up samples (51), which resulted in 199 samples.

For quantitative mass spectrometry data, we obtained protein quantification table from the PsychENCODE BrainGVEX Synapse portal (https://www.synapse.org/#!Synapse:syn5613798). This table includes abundance quantification for 11,572 proteins from 268 samples. The data processing steps for producing the mass spectrometry quantification table is detailed in Luo et al. (21). We further log-transformed protein abundance for each sample. We filtered out genes with log-transformed protein abundance < 1 in more than 75% samples and the samples with network connectivity (49) z score < −6, and found no gene and sample were filtered out. We then used the normalize.quantiles function in the R package “preprocessCore” (50) to normalize protein level for each sample. We used DRAMS software to detect and correct mixed-up samples (51), which found no mixed-up samples. We matched the protein ID to gene ID according to the UCSC database of hg19 version, which resulted in 8,330 genes.

### QTL mapping

#### Estimating and adjusting for unwanted factors

We used the R package “PEER” (54) to estimate hidden factors for RNA-Seq, ribo-seq, and proteomics data separately. The principle of selecting unwanted hidden factors was to maximize the variance explained with the least number of factors. We identified 30, 29, and 19 hidden factors to remove from RNA-Seq, ribo-seq, and mass spectrometry data, respectively (Fig. S11). For each gene from each omics type, we adjusted the expression level by fitting the selected hidden factors as predictors in a linear model and taking the residuals as the adjusted expression level. The adjusted expression levels were then further centered by mean and scaled by standard deviation.

#### Genotype association tests

We identified *cis*-region expression QTLs (*cis*-eQTLs), ribosome occupancy QTLs (*cis*-rQTLs), and protein QTLs (*cis*-pQTLs) separately using QTLTools (1.3.1) (55). Because each gene can encode several protein isoforms, we selected the protein isoform with the highest median abundance as the representative protein. For each gene, we defined *cis*-region as the region ranging from 1Mb upstream of the 5’ end of the gene to 1Mb downstream of the 3’ end of the gene (i.e. the length of the gene body plus 2 Mb in size). We tested all common SNPs (MAF > 0.05) within the *cis*-region using 10,000 permutations to create empirical null distributions and used the beta-approximation approach implemented in QTLTools to estimate the empirical p values. For each gene, we selected the SNP with the most significant empirical p value from the genotype-expression level association tests to represent the QTL signal. To calculate a genome-wide FDR, we used the qvalue function of the R package “qvalue” for multiple testing correction and set a qvalue < 0.1 (i.e. 10% FDR) cutoff to identify significant QTLs.

### Estimating mediated SCZ heritability of each omics

We used MESC to estimate the proportion of SCZ heritability mediated by different omics (32). In the first step, we calculated the overall expression score using the unwanted-factor-adjusted-expression data (see QTL mapping section) as the individual-level gene expression data with the corresponding BrainGVEX genotype data and the 1000 genome phase 3 genotype data as the ancestry matched genotype data for GWAS. In the second step, we used the overall expression score calculated in the previous step and PGC3 SCZ GWAS summary statistics to estimate SCZ heritability mediated by each omics.

### Identifying shared and omics-specific QTL genes

#### Building PrediXcan gene expression prediction models

We used the PrediXcan software (13) to separately build gene expression prediction models for RNA-Seq, ribo-seq, and proteomics. For each gene, an Elastic Net algorithm was used for feature selection from SNPs located within the *cis*-region defined for each gene (i.e. gene body +/− 1Mb flanking regions) based on results from a ten-fold cross-validation. After that, weights were produced for every selected SNP of each gene, which were used in the prediction models. For each gene, we calculated the Pearson correlation between predicted and observed gene expression (Cross-validation R, R_cv_), which was considered as a metric for prediction accuracy. Only the genes with R_cv_ > 0.1 and P < 0.05 were retained. We produced prediction models for the unified set of 7,458 genes and 185 samples shared across omics.

#### Building conditionally independent prediction models

To identify shared and omics-specific QTL genes among different omics, we built conditionally independent prediction models for each omics. For each gene, we regressed from the data the genetic regulation signals of all other omics types (i.e. the imputed quantification level of all other omics types). The models were built based on the regressed expression (i.e. the residual from the regression). Note that because in many cases multiple protein isoform quantifications match to one gene, when building model for gene-based quantification omics types, such as mRNA and ribosome occupancy, we aggregate data across all protein isoforms for each gene. In contrast, when building model for protein data, quantifications between isoforms were kept separate.

More specifically, assuming a total of *p* omics types, we took the following steps to identify shared-QTL genes and to define omics-specific QTL genes:

Step 1: For each gene and each omics type, we built original prediction models using equation 1

(1) $$E_{k}=\sum_{i=0}^{n}w_{i}S_{i}+\varepsilon ,$$

where *E*_*k*_ denotes the observed expression of omics type *k*; *w*_*i*_ denotes the weight of the *i*-th SNP *S*_*i*_; n denotes the number of SNPs selected by the elastic net.

We can calculate the imputed expression with genotype data: ${\hat{E}}_{k}=\sum_{i=0}^{n}w_{i}S_{i}$.

Step 2: For each gene in omics type *k*, we regressed out the imputed expression on the same gene of all the other omics types from the observed expression using equation 2 and kept the residual

(2) $$E_{k}=\beta_{1}\hat{E_{1}}+\beta_{2}\hat{E_{2}}+\cdots +\beta_{l-1}\hat{E_{l-1}}+e_{k},$$

where *β*_*l*_ denotes slope of the *l*-th other omics types, ${\hat{E}}_{l}$ denotes the imputed expression of the *l*-th other omics types, and *e*_*k*_ denotes the residual for omics type *k*. Then, we calculated the expression value conditioning on the genetic regulation of all the other omics types: $R_{k}=\bar{E_{k}}+e_{k}$. Where $\bar{E_{k}}$ is the sum of mean expression across genes.

Step 3: After that, we built the conditional prediction models and estimated the SNP-weights parameters) using equation 3.

(3) $$R_{k}=\sum_{i=0}^{n}m_{i}S_{i}+\varepsilon ,$$

where *m*_*i*_ is the weight of *i*-th SNP *S*_*i*_ for the conditional prediction model.

Step 4: We calculated the square of correlation of imputed gene expression between the original model and conditional model and named it as observed R_c_^2^.

Step 5: To determine whether a gene share genetic regulation signals with other omics types, we used permutation to create a null distribution of R_c_^2^. We permuted the sample label for imputed expression of the other omics types, and repeated step 2to build the conditional prediction model using permuted data, which we named conditional permutation model. We then calculated permutation R_c_^2^ of imputed gene expression between the original model and conditional permutation model.

Step 6: We performed 50 permutations and calculated empirical p values of the observed R_c_^2^ based on its rank among the permutation R ^2^_c_ (i.e. ordered from the smallest to the greatest).

Step 7: We used Benjamini-Hochberg control procedure to adjust the empirical p values to calculate FDR for shared-QTL genes.

Step 8: We defined omics-specific QTL genes as genes with FDR > 0.1 and R_c_^2^ >0.9, and the omics-shared QTL genes as genes with FDR <0.1 and R_c_^2^ <0.5.

### Colocalization

We used *coloc* (11) to detect signal colocalization between SCZ GWAS and each QTL type at the cis-region of each QTL gene. For each QTL gene, for all common SNPs (MAF > 0.05) within the cis-region, we use QTL effects and GWAS summary statistics as input for coloc analysis. For QTL effects, we calculated slope and square of slope’ standard error from a linear model fit using SNP genotype as the predictor of gene quantifications. We then used the coloc.abf function in the R package “*coloc*” (11) to calculate the posterior possibility of each hypothesis using the default prior. We use posterior probability of 70% for the colocalization hypothesis (i.e. PPH4) as the cutoff for reporting our colocalization findings.

### TWAS identification of SCZ risk genes

Gene-level association tests were performed for SCZ using S-PrediXcan (33) based on the prediction models built using our omics data, which is described in the “building prediction models” section, and the SCZ GWAS summary statistics data from PGC3 (17). The association tests were done separately for each omics. For protein data, we performed omnibus test to incorporate p values of all protein isoforms together to calculate a single p value for the corresponding gene. The family wise error rates for SCZ risk genes were calculated using Bonferroni correction of nominal p values.

### Two-sample Mendelian randomization (MR)

To identify causal relationships between each omics type and SCZ we used MR analysis. Here we used fine mapped QTL SNPs as instruments, gene expression quantification at each omics type as exposure, and SCZ GWAS signal as outcome.

More specifically, we took the following steps to test for causal relationship between gene regulation at each omics level and SCZ:

Step 1: Here we used two methods of selecting instrument SNPs. For each gene of each QTL type, we performed LD clumping by PLINK with “–clump-kb 1000 –clump-r2 0.5” parameters to select instrument SNPs with p value < 0.05. Another method is fine-mapping by susie_rss() function with default parameters in R package “SuSiE” (44).

Step 2: For each gene, we used harmonise_data() function in R package “TwoSampleMR” (56) to harmonize QTLs of each omics type and SCZ GWAS SNPs to be in the same direction (i.e. effect relative to the same allele).

Step 3: We then performed two-sample MR for each gene in each omics type separately. Two-sample MR analysis was done using mr() function, which includes IVW and Egger methods, in the R package “TwoSampleMR” (56).

Step 4: We used the intercept test (i.e. the Egger method) to test for horizontal pleiotropy, and used predictor coefficient and its corresponding p value from IVW to determine the effect size and significance of causal effects for each omics type on SCZ.

Step 5: We used Benjamini-Hochberg to adjust for multiple testing.

Step 6: We define a gene by omics type combination as causal for SCZ if the causal effect test passed the FDR < 0.1 cutoff and the Egger intercept test passed the intercept p value > 0.05 cutoff.

### One-sample Mendelian randomization

For one-sample MR we used two-stage least squares (2SLS) approach to find causal relationships between omics types. We performed the following analysis in two iterations, both following the direction of genetic information flow. In the first iteration, we tested causal relationships between transcript level and ribosome occupancy level (i.e. mRNA -> ribosome occupancy). In the second iteration, we tested causal relationships between ribosome occupancy level and protein level (i.e. ribosome occupancy -> protein).

Step 1: We used the same two methods for instrument SNP selection as described in the previous section. Here we tested two pathways (mRNA -> ribosome occupancy and ribosome occupancy - > protein). For mRNA -> ribosome occupancy, we used the SNPs of eQTL with p value < 0.05 and LD clumping by PLINK with “–clump-kb 1000 –clump-r2 0.5” parameters. For ribosome occupancy -> protein, we used the SNPs of rQTL with p value < 0.05 and LD clumping by PLINK with “–clump-kb 1000 –clump-r2 0.5”.

Step 2: We then combined genotype and quantification data of the relevant omics types into a dataframe: dat_mr_ for mRNA -> ribosome occupancy pathway, dat_rp_ for ribosome occupancy -> protein pathway.

Step 3: For each gene, we then set formula in R package “ivreg”: ivreg(“ribosome occupancy ~ mRNA | SNP_1_+SNP_2_+SNP_3_+…+SNP_n_”, data = dat_mr_) and ivreg(“protein ~ ribosome occupancy | SNP_1_+SNP_2_+SNP_3_+…+SNP_m_”, data = dat_rp_).

Step 4: We used the summary() function to get slope, p value of slope, intercept, p value of intercept, F-statistic and p value of F-statistic of each ivreg object. P value of Intercept was used to test horizontal pleiotropy, F-statistic was used to check instrument strength.

Step 5: We used Benjamini-Hochberg control procedure to adjust for multiple testing.

Step 6: We defined a causal relationship for each gene by pathway combination as the causal effect test passed the FDR < 0.1 cutoff and the Egger intercept test passed the intercept p value > 0.05 cutoff.

## Data and materials availability:

All raw data from psychENCODE BrainGVEX project are described in https://www.synapse.org/#!Synapse:syn5613798. All code, materials and results used in the analyses are available in https://www.synapse.org/#!Synapse:syn51664324. All data are available in the main text or the supplementary materials.

## Supplementary Material

Supplement 1

Supplement 2
