# Supplementary material for: The impact of common variants on gene expression in the human brain: from RNA to protein to schizophrenia risk
Source: bioRxiv. 2023 Oct 10:2023.06.04.543603. Preprint. [Version 3] doi: 10.1101/2023.06.04.543603 (PMC10592607; doi:10.1101/2023.06.04.543603)
Supplement: Supplement 1 [file media-1.docx]

**Supplementary Materials**


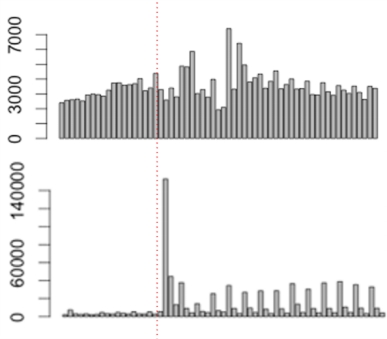


**Fig. S1.** **Example sub-codon periodicity pattern of ribosome footprints.** Sequencing read counts were aggregated across genes according to the distance between the 5’ end of each mapped read and the annotated translation initiation sites of the gene it mapped to. Top panel: RNA-Seq data. Bottom panel: ribo-seq data. The red dotted line indicates the 5’ end of the translation initiation site. X-axis: relative distance to the translation initiation site, one nucleotide per bar. Y-axis: number of reads aggregated at each relative position.


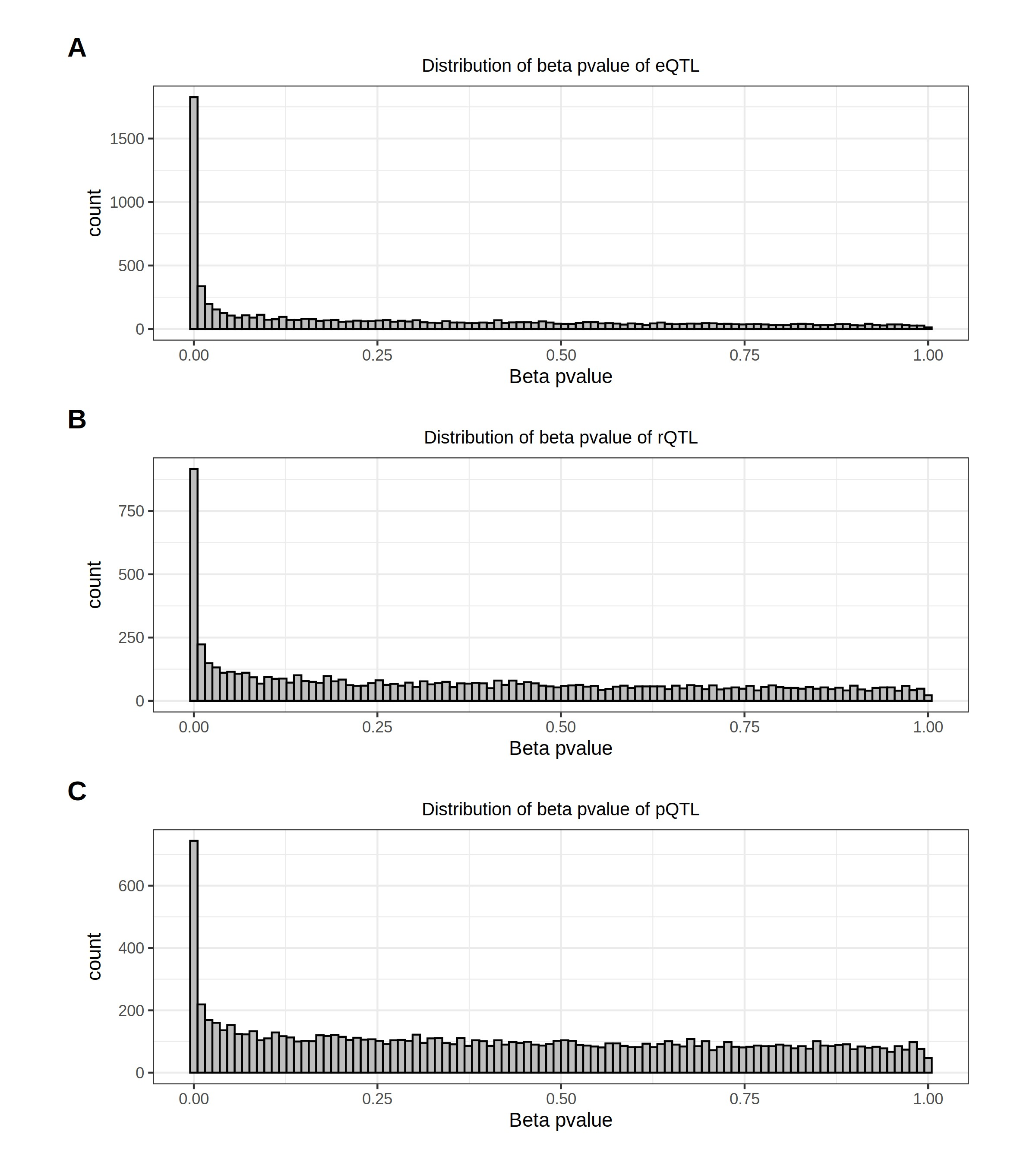


**Fig. S2. Distribution plots of beta pvalues of permutation QTLs at each level. (A)** Histogram of pvalues of eQTL. **(B)** Histogram of pvalues of rQTL. **(C)** Histogram of pvalues of pQTL.


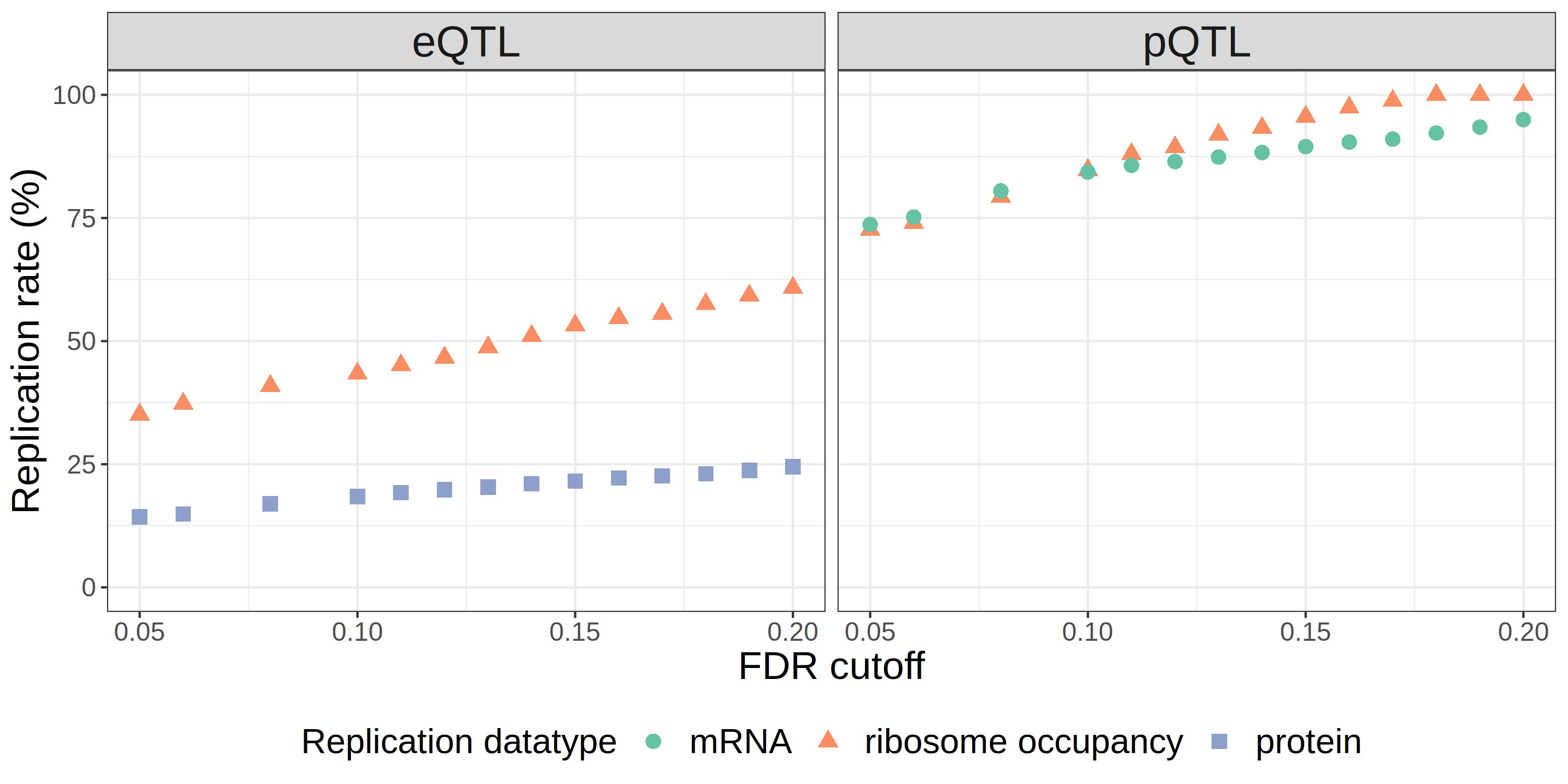


**Fig. S3.** **Replication rates between QTL types.** The discovery QTL types are labeled at the top. The replication datatypes are color and shape coded with the color and shape key positioned to the bottom of the plots. X-axis: the FDR cutoff used to define QTL replication (i.e. from 5% to 20%). Y-axis: percentage of QTLs replicated (i.e. from 0 to 100%).

**
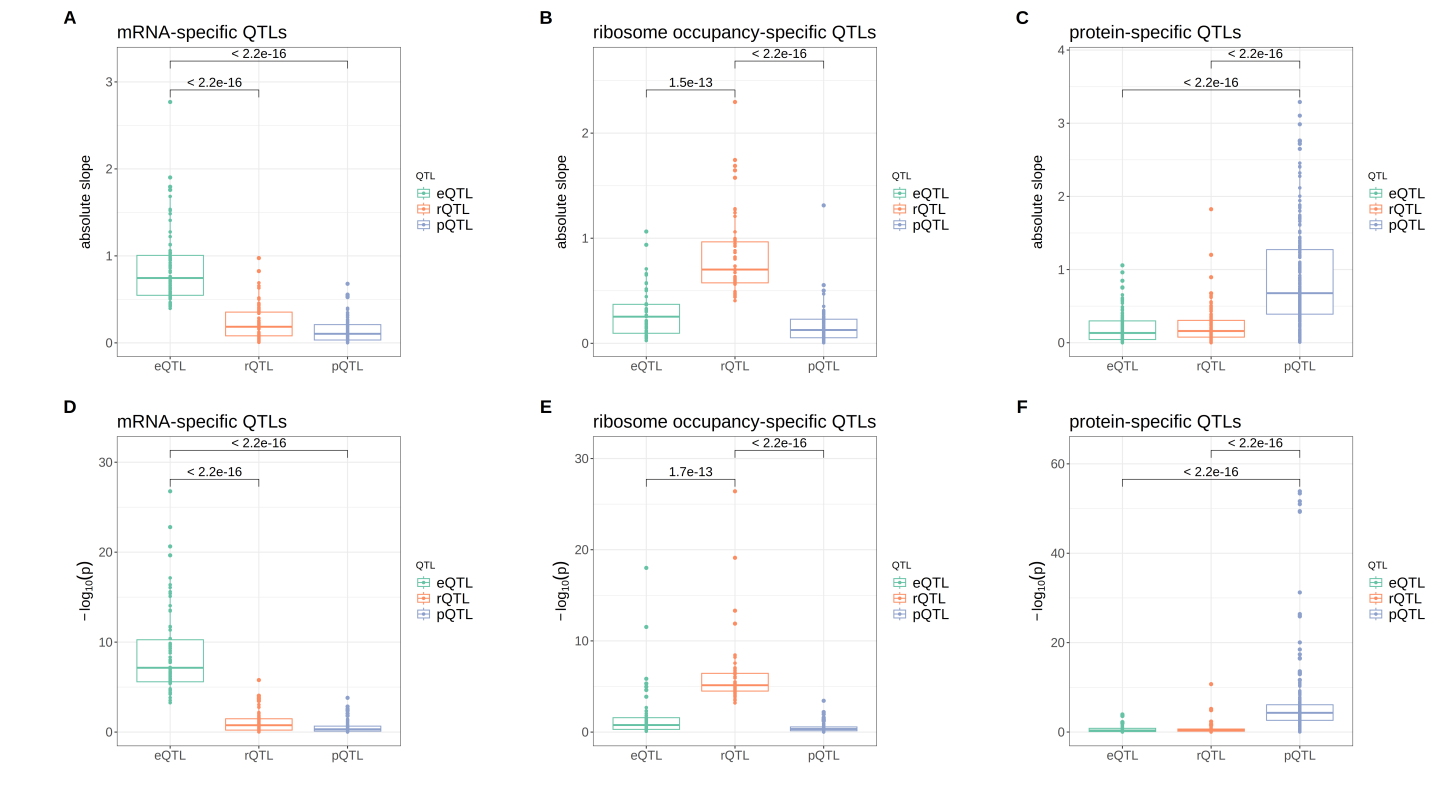
**

**Fig. S4.** **Datatype-specific QTLs show expected patterns in effect size and p value across datatypes.** **(A, B, C)** Boxplots summarizing the absolute slope and **(D, E, F)** Boxplots summarizing the corresponding -log_10_p value associated with datatype specific QTL genotypes in a linear model fit of expression level from each datatype.


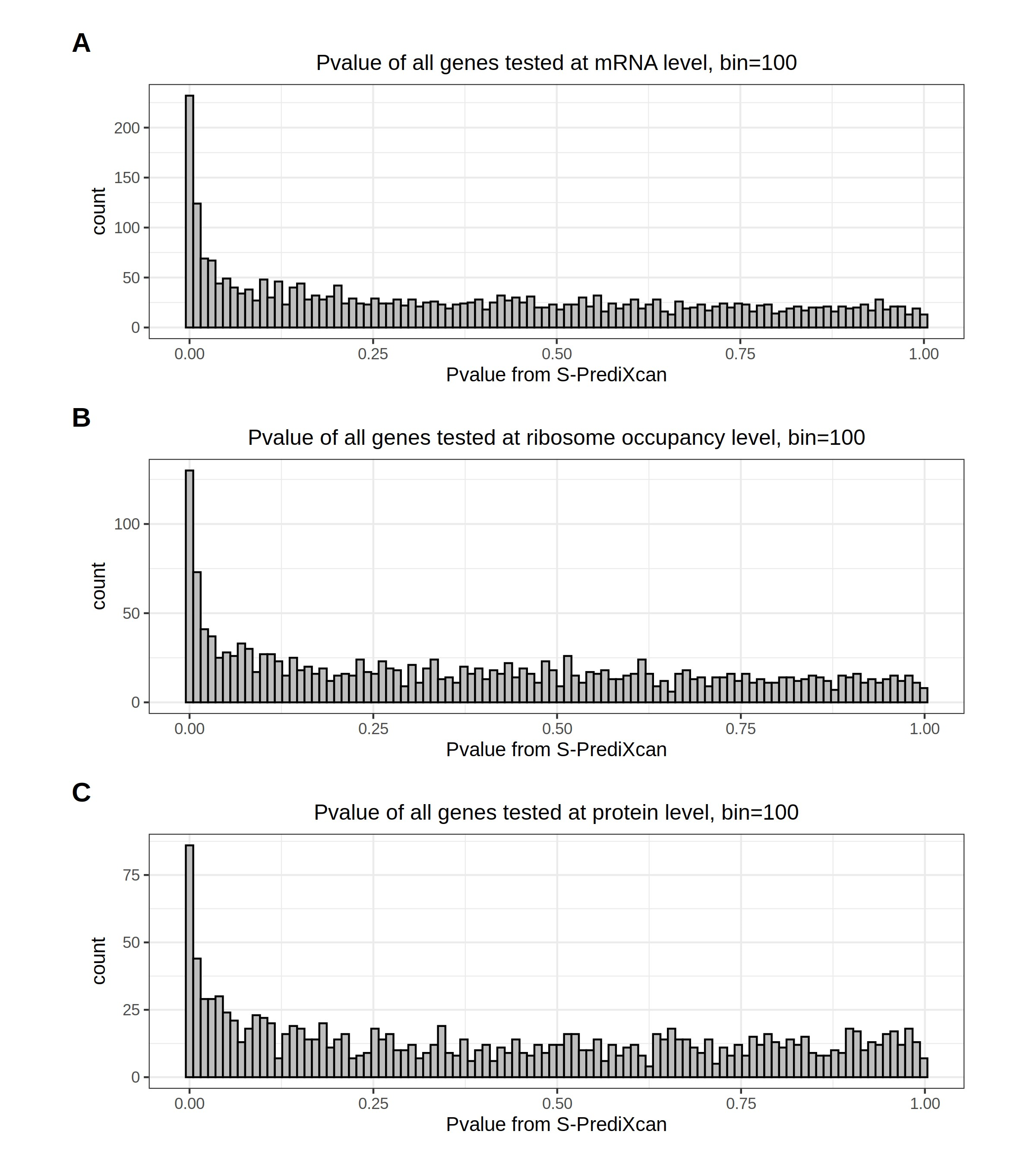


**Fig. S5. Distribution of pvalues of all genes when detecting SCZ risk genes using S-PrediXcan. (A)** Histogram of pvalues at mRNA level. **(B)** Histogram of pvalues at ribosome occupancy level. **(C)** Histogram of pvalues at protein level.


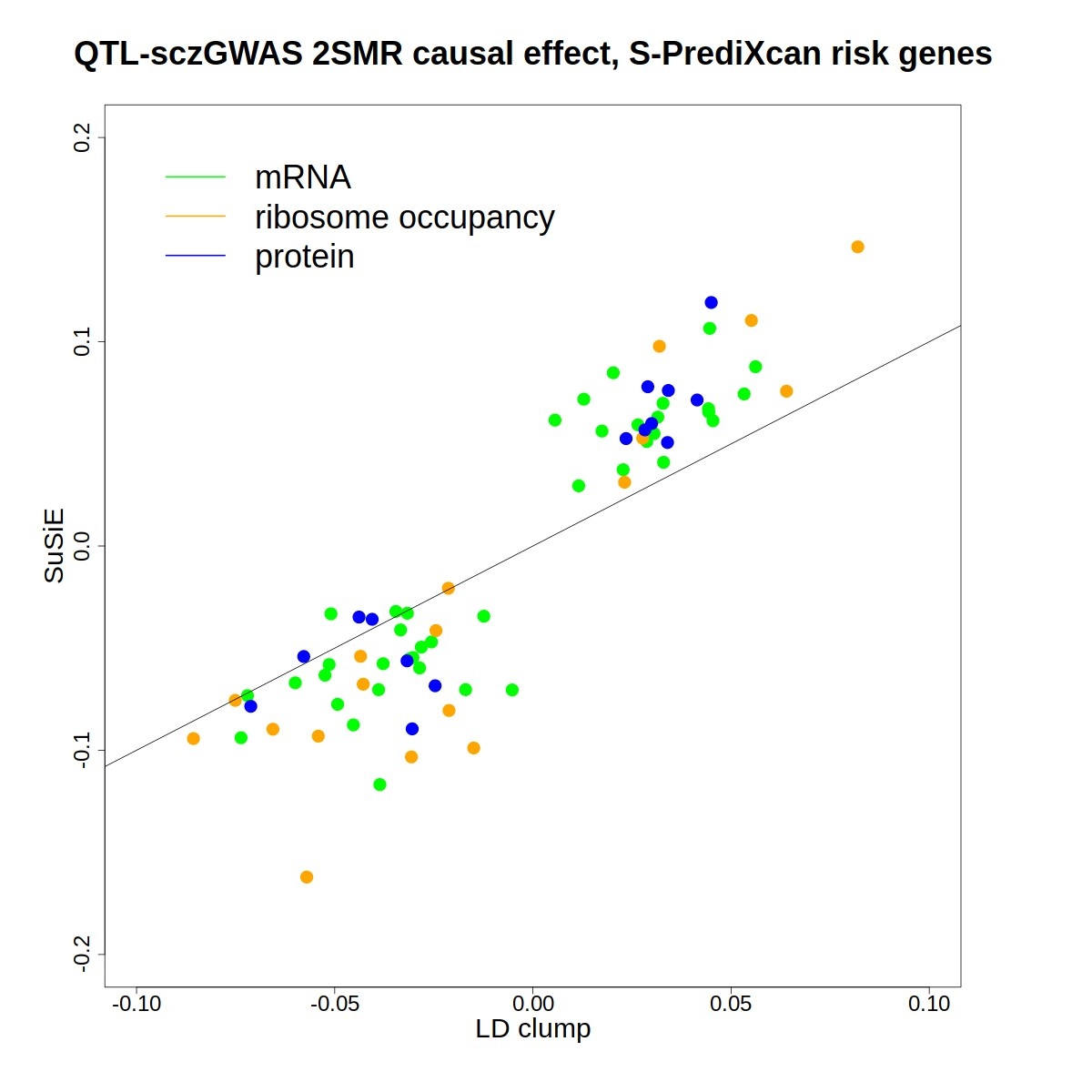


**Fig. S6.** **Scatter plot of SCZ risk gene QTL causal effects estimated via two-sample MR using two different methods of selecting instrument SNPs.** Each dot represents a risk gene causal effect on schizophrenia GWAS signal (i.e. the slope associated with risk gene QTLs) calculated from two-sample MR using LD clumped SNPs (X-axis) vs. SuSiE fine-mapped SNPs (Y-axis) as instrument variable.


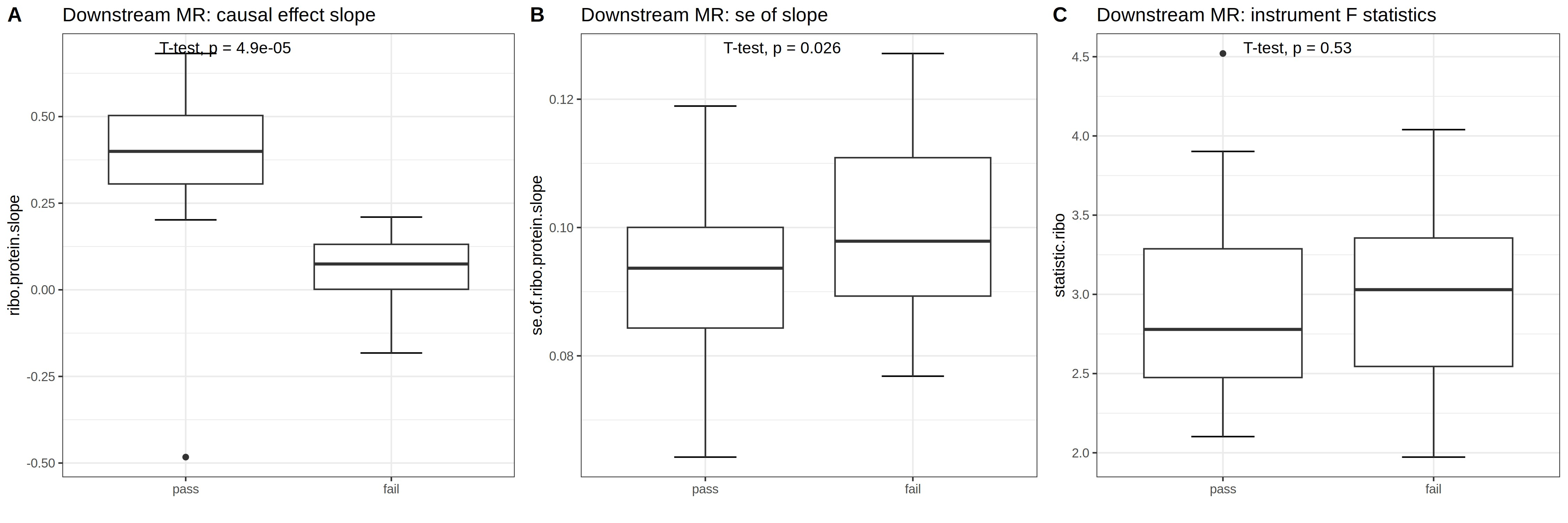


**Fig. S7.** **Boxplots summarizing coefficients and statistics of the downstream pathway one-sample-MR tests for upstream-passed genes (fail) versus both-passed genes (pass).** **(A)** coefficient (slope) associated with the predictor of the MR tests. **(B)** standard error of the slope. **(C)** F statistics from the exposure-instrument variable regression.


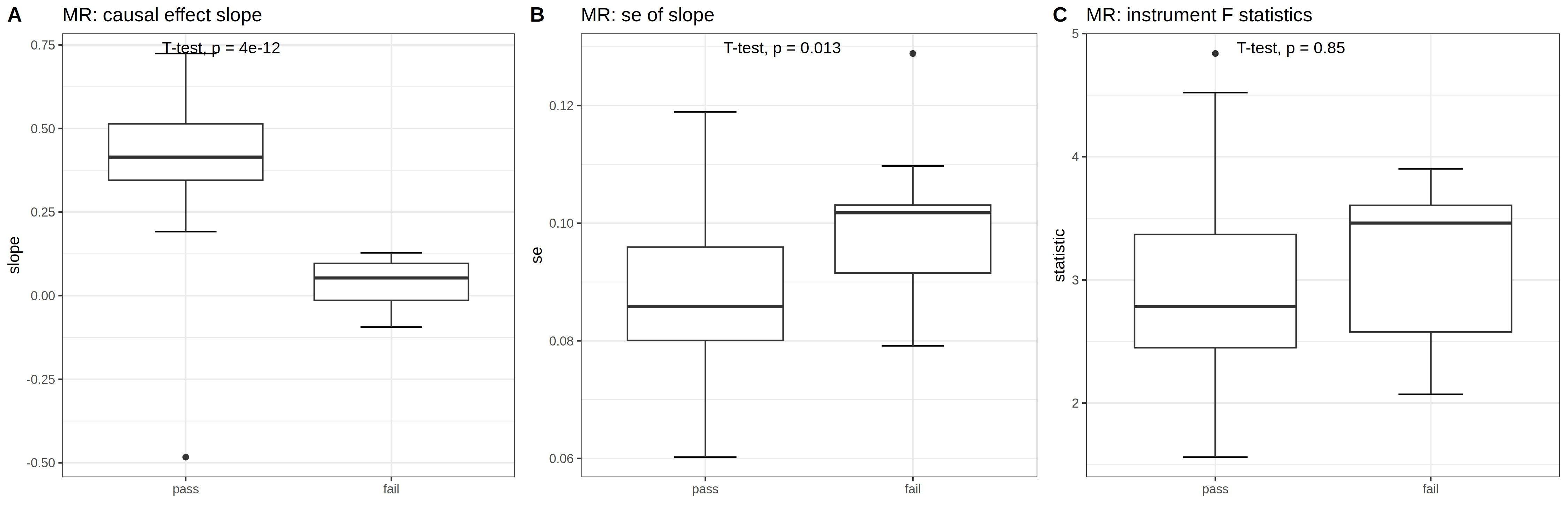


**Fig. S8.** **Boxplots summarizing coefficients and statistics of the one-sample-MR tests for none-passed genes (fail) versus both-passed genes (pass).** **(A)** coefficient (slope) associated with the predictor of the MR tests. **(B)** standard error of the slope. **(C)** F statistics from the exposure-instrument variable regression.

**
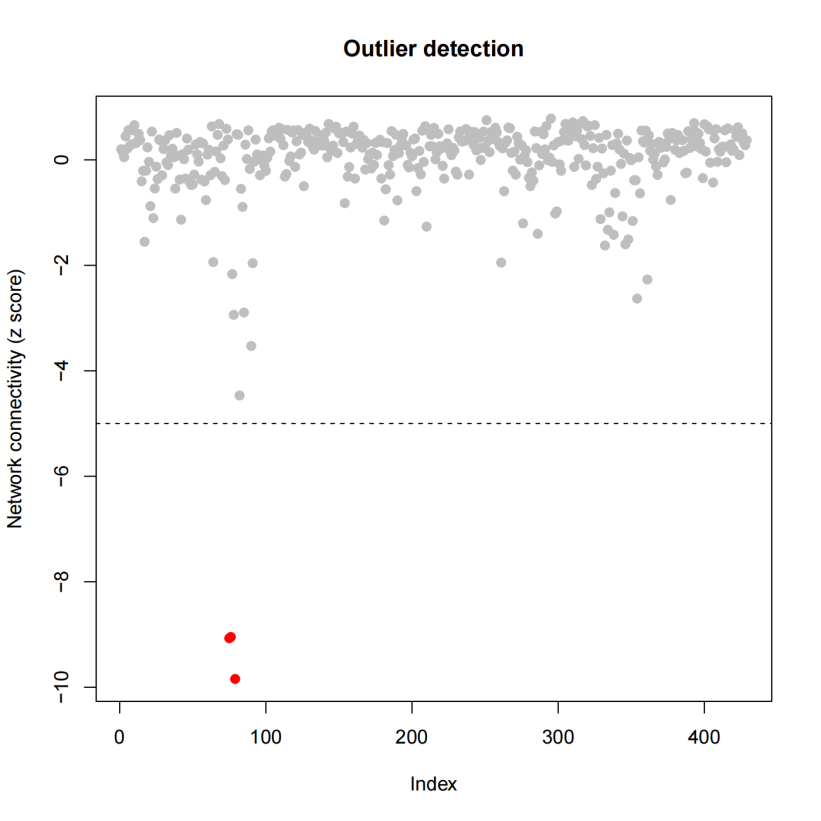
**

**Fig. S9.** **Plot of network connectivity z score of RNA-Seq gene expression.** Each dot represents a sample. The red dots denote the samples excluded from the QTL analysis.


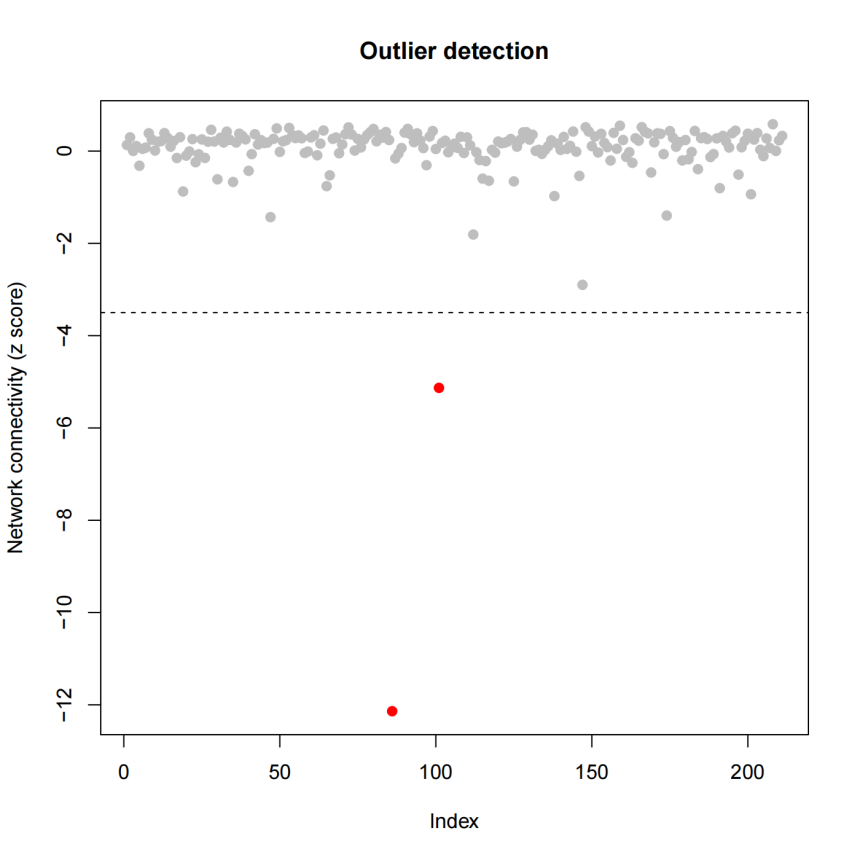


**Fig. S10.** **Plot of network connectivity z score of ribo-seq gene expression.** Each dot represents a sample. The red dots denote the samples excluded from the QTL analysis.


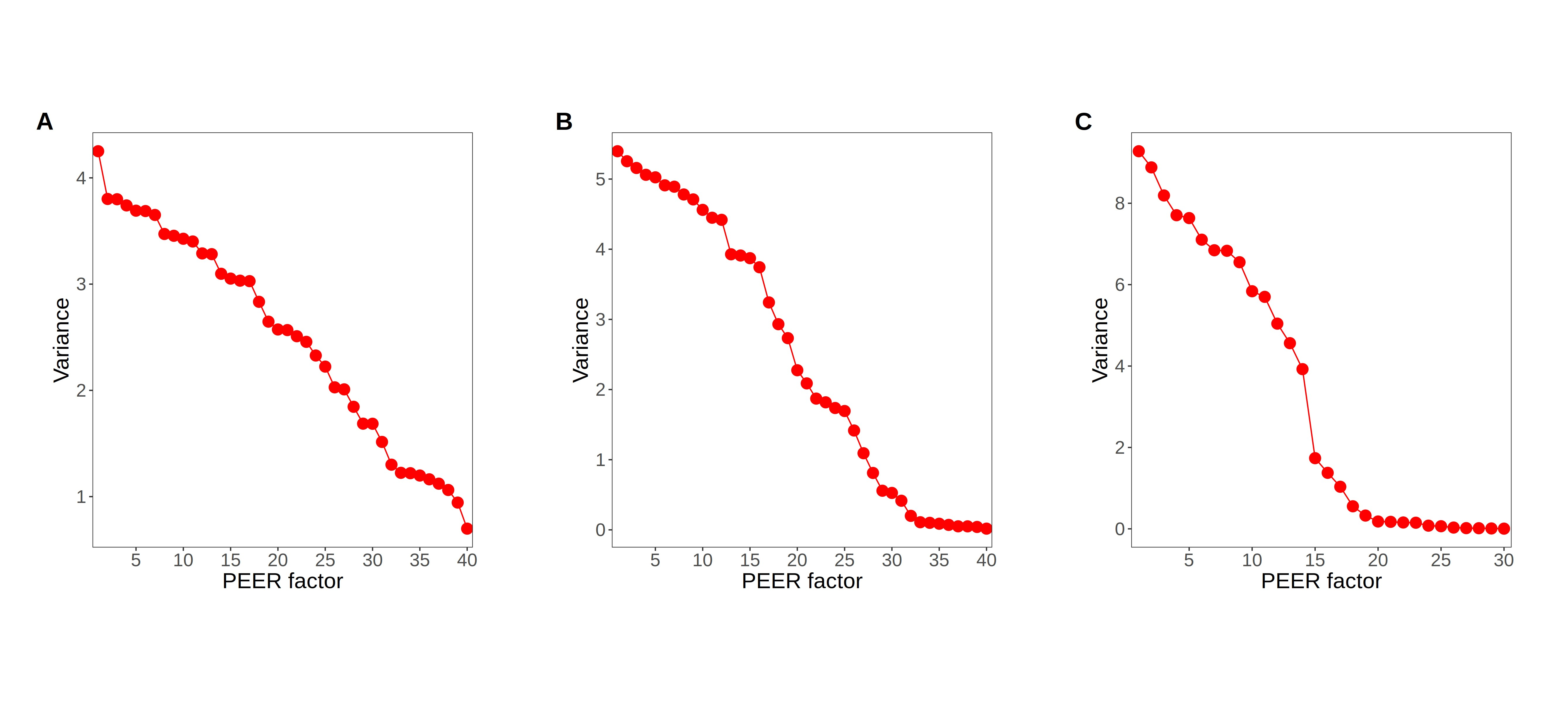


**Fig. S11. Variance explained by PEER factors. (A)** RNA-Seq. **(B)** Ribo-seq. **(C)** Quantitative mass spectrometry.

**PsychENCODE Consortium Authors and Affiliations**

‡ Schahram Akbarian^1^, Alexej Abyzov^2^, Nadav Ahituv^3^, Dhivya Arasappan^4^, Jose Juan Almagro Armenteros^5^, Brian Beliveau^6^, Jaroslav Bendl^1^, Sabina Berretta^7^, Rahul Bharadwaj^8^, Arjun Bhattacharya^9^, Lucy Bicks^9^, Kristen Brennand^10^, Davide Capauto^10^, Frances A. Champagne^4^, Tanima Chatterjee^10^, Christos Chatzinakos^7^, Yuhang Chen^10^, Han-Chia Chen^11^, Yuyan Cheng^9^, Lijun Cheng^12^, Andrew Chess^1^, Jo-fan Chien^13^, Zhiyuan Chu^10^, Declan Clarke^10^, Ashley Clement^3^, Leonardo Collado-Torres^8^, Gregory Cooper^14^, Gregory Crawford^15^, Rujia Dai^16^, Nikolaos P. Daskalakis^7^, Jose Davila-Velderrain^17^, Amy Deep^8^, Chengyu Deng^3^, Chris DiPietro^7^, Stella Dracheva^1^, Shiron Drusinsky^18^, Ziheng Duan^19^, Duc Duong^20^, Cagatay Dursun^10^, Nick Eagles^8^, Jonathan Edelstien^1^,Prashant S. Emani^10^, John Fullard^1^, Kiki Galani^21^, Timur Galeev^10^, Michael J. Gandal^11^, Sophia Gaynor^12^, Mark Gerstein^10^, Daniel Geschwind^9^, Kiran Girdhar^1^, Fernando S. Goes^22^, William Greenleaf^5^, Jennifer Grundman^9^, Qiuyu Guo^9^, Chirag Gupta^23^, Yoav Hadas^1^, Joachim Hallmayer^5^, Xikun Han^21^, Vahram Haroutunian^1^, Natalie Hawken^9^, Chuan He^24^, Ella Henry^10^, Joo Heon Shin^8^, Stephanie Hicks^8^, Marcus Ho^5^, Li-Lun Ho^21^, Gabriel E. Hoffman^1^, Yiling Huang^5^, Louise Huuki^8^, Ahyeon Hwang^19^, Thomas Hyde^8^, Artemis Iatrou^7^, Fumitaka Inoue^3^, Aarti Jajoo^7^, Matthew Jensen^10^, Lihua Jiang^5^, Peng Jin^20^, Ting Jin^23^, Connor Jops^11^, Alexandre Jourdon^10^, Riki Kawaguchi^9^, Manolis Kellis^21^, Joel Kleinman^8^, Steven P. Kleopoulos^1^, Alex Kozlenkov^1^, Arnold Kriegstein^3^, Anshul Kundaje^5^, Soumya Kundu^5^, Cheyu Lee ,University California Irvine^19^, Donghoon Lee^1^, Junhao Li^13^, Mingfeng Li^10^, Xiao Lin^1^, Shuang Liu^10^, Jason Liu^10^, Jianyin Liu^9^, Chunyu Liu^16^, Shuang Liu^23^, Shaoke Lou^10^, Jacob Loupe^14^, Dan Lu^25^, Shaojie Ma^10^, Liang Ma^26^, Michael Margolis^9^, Jessica Mariani^10^, Keri Martinowich^8^, Kristen R. Maynard^8^, Samantha Mazariegos^9^, Ran Meng^10^, Richard Meyers^14^, Courtney Micallef^1^, Tatiana Mikhailova^16^, Guo-li Ming^11^, Shahin Mohammadi^27^, Emma Monte^5^, Kelsey S. Montgomery^25^, Jill E. Moore^28^, Jennifer Moran^12^, Eran Mukamel^13^, Angus Nairn^10^, Charles Nemeroff^29^, Pengyu Ni^10^, Scott Norton^10^, Tomasz Nowakowski^3^, Larsson Omberg^25^, Stephanie C. Page^8^, Saejeong Park^10^, Ashok Patowary^9^, Reenal Pattni^5^, Geo Pertea^8^, Mette A. Peters^25^, Nishigandha Phalke^28^, Dalila Pinto^1^, Milos Pjanic^1^, Sirisha Pochareddy^10^, Katherine Pollard^18^, Alex Pollen^3^, Henry Pratt^28^, Pawel F. Przytycki^18^, Carolin Purmann^5^, Zhaohui S. Qin^20^, Ping-Ping Qu^5^, Diana Quintero^9^, Towfique Raj^1^, Ananya S. Rajagopalan^10^, Sarah Reach^1^ , Thomas Reimonn^28^, Kerry J. Ressler^7^, Deanna Ross^4^, Panagiotis Roussos^1^, Joel Rozowsky^10^, Misir Ruth^1^, W. Brad Ruzicka^7^, Stephan J. Sanders^30^, Juliane M. Schneider^25^, Soraya Scuderi^10^, Robert Sebra^1^, Nenad Sestan^10^, Nicholas Seyfried^20^, Zhiping Shao^1^, Nicole Shedd^28^, Annie W. Shieh^31^, Mario Skarica^10^, Clara Snijders^7^, Hongjun Song^11^, Matthew State^3^, Jason Stein^32^, Marilyn Steyert^3^, Sivan Subburaju^7^, Thomas Sudhof^5^, Michael Synder^5^, Ran Tao^8^, Karen Therrien^1^, Li-Huei Tsai^21^, Alexander Urban^5^, Flora M. Vaccarino^10^, Harm van Bakel^1^, Daniel Vo^11^, Georgios Voloudakis^1^, Brie Wamsley^9^, Tao Wang^5^, Sidney H. Wang^31^, Daifeng Wang^23^, Yifan Wang^2^, Jonathan Warrell^10^, Yu Wei^16^, Annika Weimer^5^, Daniel R. Weinberger^8^, Cindy Wen^9^, Zhiping Weng^28^, Sean Whalen^18^, Kevin White^33^, A Jeremy. Willsey^3^, Hyejung Won^32^, Wing Wong^5^, Hao Wu^20^, Feinan Wu^10^, Stefan Wuchty^34^, Dennis Wylie^4^, Siwei Xu^19^, Chloe X. Yap^34^, Biao Zeng^1^, Pan Zhang^9^, Chunling Zhang^16^, Bin Zhang^1^, Jing Zhang^19^, Yanqiong Zhang^32^, Xiao Zhou^10^, Ryan Ziffra^3^, Trisha M. Zintel^25^

^1^Icahn School of Medicine at Mount Sinai, New York, NY, USA. ^2^Mayo Clinic Rochester, Rochester, MN, USA. ^3^University of California, San Francisco, San Francisco, CA, USA. ^4^The University of Texas at Austin, Austin, TX, USA. ^5^Stanford University, Stanford, CA, USA. ^6^University of Washington, Seattle, WA, USA. ^7^McLean Hospital, Belmont, MA, USA. ^8^Lieber Institute for Brain Development, Baltimore, MD, USA. ^9^University of California, Los Angeles, Los Angeles, CA, USA. ^10^Yale University, New Haven, CT, USA. ^11^University of Pennsylvania, Philadelphia, PA, USA. ^12^Tempus Labs, Inc., Chicago, IL, USA. ^13^University of California, San Diego, San Diego, CA, USA. ^14^HudsonAlpha Institute for Biotechnology, Huntsville, AL, USA. ^15^Duke University, Durham, NC, USA. ^16^SUNY Upstate Medical University, Syracuse, NY, USA. ^17^Human Technopole, Milan, Italy. ^18^Gladstone Institutes, University of California, San Francisco, San Francisco, CA, USA. ^19^University of California, Irvine, Irvine, CA, USA. ^20^Emory University, Atlanta, GA, USA. ^21^Massachusetts Institute of Technology, Cambridge, MA, USA. ^22^Johns Hopkins University, Baltimore, MD, USA. ^23^University of Wisconsin-Madison, Madison, WI, USA. ^24^The University of Chicago, Chicago, IL, USA. ^25^Sage Bionetworks, Seattle, WA, USA. ^26^The University of Texas Health Science Center at San Antonio, San Antonio, TX, USA. ^27^Broad Institute of MIT and Harvard, Cambridge, MA, USA. ^28^University of Massachusetts Chan Medical School, Worcester, MA, USA. ^29^The University of Texas at Austin Dell Medical School, Austin, MA, USA. ^30^University of Oxford, Oxford, England, UK. ^31^The University of Texas Health Science Center at Houston, Houston, TX, USA. ^32^University of North Carolina at Chapel Hill, Chapel Hill, USA. ^33^National University of Singapore, Singapore, Singapore. ^34^University of Miami, Miami, FL, USA. University of Queensland, Queensland, NZ.
